# **Building a directed evolution**–**genome editing pipeline for metabolic traits in specialty crop breeding**

**DOI:** 10.1093/hr/uhaf203

**Published:** 2025-10-25

**Authors:** Anuran K Gayen, Laura M Carmona Rojas, Zhen Fan, Seonghee Lee, Vance M Whitaker, Andrew D Hanson

**Affiliations:** Horticultural Sciences Department, University of Florida, Gainesville, FL 32611-0690, USA; Horticultural Sciences Department, University of Florida, Gainesville, FL 32611-0690, USA; Horticultural Sciences Department, Institute of Food and Agricultural Sciences (IFAS) Gulf Coast Research and Education Center, University of Florida, Wimauma, FL 33598-6101, USA; Horticultural Sciences Department, Institute of Food and Agricultural Sciences (IFAS) Gulf Coast Research and Education Center, University of Florida, Wimauma, FL 33598-6101, USA; Horticultural Sciences Department, Institute of Food and Agricultural Sciences (IFAS) Gulf Coast Research and Education Center, University of Florida, Wimauma, FL 33598-6101, USA; Horticultural Sciences Department, University of Florida, Gainesville, FL 32611-0690, USA

##  

CRISPR/Cas genome editing (GE) technology has opened unprecedented opportunities for breeding specialty and commodity crops [1–3]. The advent of base editors and prime editors is enabling multiple precise edits within a gene, which have far more potential than the imprecise insertions or deletions (indels) made by first-generation GE technology. Indels typically just knock out genes or gene domains [1, 2]. Examples of such loss-of-function GE in a specialty crop (tomato) are raising sugar content by knocking out two protein kinases [4] and raising γ-aminobutyrate (GABA) content by deleting the autoinhibitory domain of glutamate decarboxylases [5]. In contrast, being able to introduce specific sequence changes makes it possible to craft change-of-function or gain-of-function mutations, e.g. to improve an enzyme’s kinetics or equip it to act on a new substrate [6, 7]. Deploying such next-generation GE is a priority because the number of target traits that can be improved by first-generation GE knockouts is limited to begin with, and such targets are being steadily picked off [1, 2].

Because crafting change- or gain-of-function mutations requires knowing not only which gene(s) to target but also *exactly which edits to make*; this exact knowledge is key to the future of GE in breeding. Directed evolution (DE) technology, informed by biochemistry, can deliver this knowledge. In brief, DE uses a fast-throughput microbial platform to hypermutate an enzyme or other target gene and selects mutations that confer a desired characteristic, e.g. activity with a new substrate [6, 8]. Sequencing the evolved gene reveals the mutations that improved the characteristic (often six or fewer) [6, 9]. DE thus specifies the mutations for GE to make. Information about metabolic biochemistry and its impacts on phenotypes guides which target enzyme to choose and the characteristic to select for [10, 11]. The DE–GE combination is thus a synergistic pipeline, far more powerful than DE or GE alone. In essence, this pipeline takes a gene out of the plant, improves it, and puts the improved gene back (Fig. 1). Because DE–GE can in principle be applied to almost any enzyme in plant primary or secondary metabolism, it is a powerful way to add variation – synthetic variation – to crop gene pools for metabolic traits. Such traits span the range from photosynthetic efficiency [12] to natural product profiles [13].

**Figure 1:**
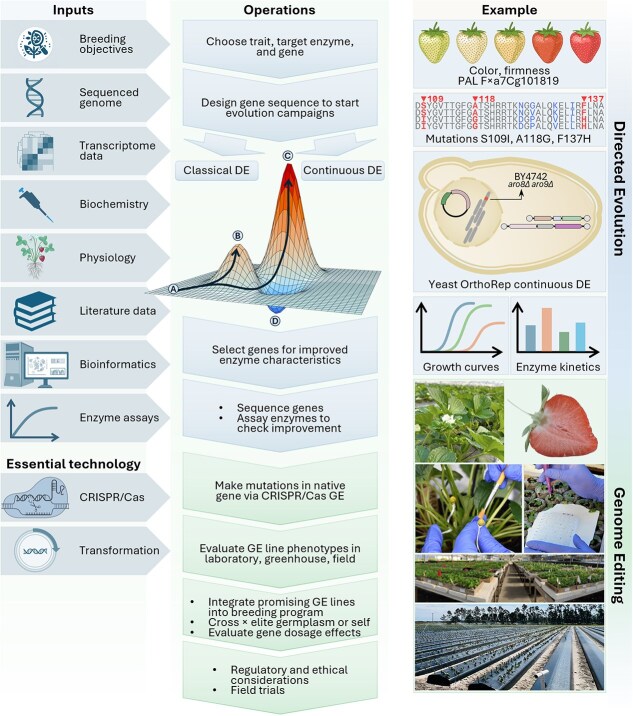
The directed evolution–genome editing (DE–GE) pipeline shows the sequence of steps from target choice to breeding germplasm in the center, with the associated knowledge and resource inputs on the left and the example of PAL in strawberry fruit schematized on the right. The schematic fitness landscape in the center shows the starting point for directed evolution at A, fitness peak B that is readily reached via an uninterrupted series of mutations, and fitness peak C that can only be reached by first crossing fitness valley D.

As the DE part of the pipeline, including choice of target enzyme, has had essentially no coverage in the breeding literature on GE other than the original description of DE–GE [10] and reviews that recapitulate the concept(e.g. [11, 14]), this perspective explains DE, particularly its first steps, and how DE can interface with GE in a breeding program. We illustrate each step in the pipeline using the enzyme phenylalanine ammonia-lyase (PAL) in strawberry fruit. PAL is a suitable target for DE–GE because it underlies important fruit quality traits, including anthocyanin content and lignin content [15, 16]. Note that a DE–GE pipeline can in principle be implemented in any case where a target enzyme has been identified and linked to a metabolic trait, and where genome editing is established. Crops such as apple, cotton, maize, soybean, banana, tomato, rice, potato, grape, watermelon, cucumber, and citrus are all good candidates for applying the DE–GE strategy to various metabolic traits [2, 17].

## What directed evolution does

The tracks in the fitness landscape image at the core of Fig. 1 show how DE recapitulates natural evolution by stepwise selection of mutations that improve a gene’s performance, i.e. that climb a fitness peak [6, 8]. The difference from natural evolution is the speed of progress due to the extremely high mutation rate and highly focused selection made possible by using a microbe (usually *Escherichia coli* or yeast) as the platform. For instance, mutation rates can be up to 1 million times the natural rate [18] and DE can in principle do in months what could take classical breeding thousands of years [19].

**Figure 2:**
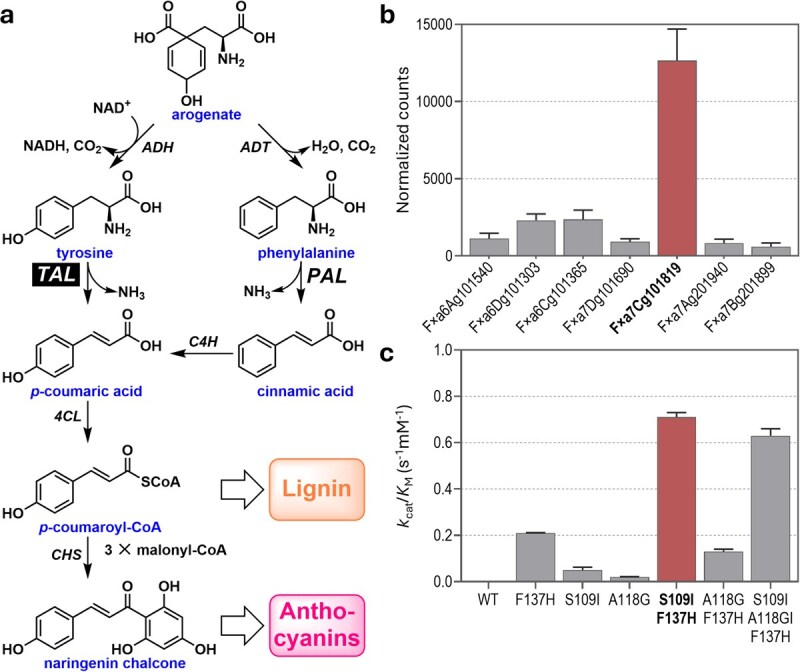
Selecting the target gene and optimizing it for directed evolution. (a) The phenylpropanoid pathway and the extra route from arogenate to *p*-coumarate that adding TAL activity installs. Abbreviations: ADH, arogenate dehydrogenase; ADT, arogenate dehydratase; CHS, chalcone synthase; 4CL, *p*-coumarate:CoA ligase; PAL, phenylalanine ammonia-lyase; TAL, tyrosine ammonia-lyase. (b) Expression in strawberry fruit of the seven PAL genes. Values are means and SE (*n* = 6). (c) Catalytic efficiency (*k*_cat_/*K*_M_) of wildtype and mutant FxaPAL1 enzymes with tyrosine as substrate. Values are means and SE (*n* = 3). See Table S1 for further kinetic analysis and see Supplementary Information for methods used to analyze gene expression enzyme activity.

There are two basic types of DE: classical and continuous (Fig. 1). Neither type depends on knowledge about the target enzyme’s 3D structure or catalytic mechanism, giving them an advantage over rational design, which requires such knowledge and hence can be applied only to well-studied enzymes [20]. Classical DE—for which Frances Arnold received the 2018 Nobel Prize in Chemistry [21]—typically introduces mutations into an enzyme gene *in vitro*, e.g. by error-prone PCR, transforms the resulting library into platform cells, then expresses and extracts the enzyme and assays it *in vitro*. Improved mutants are thereby identified, then entered into further cycles of mutation, expression, and screening assays until the desired improvement is reached. This process is labor-intensive and consequently limited in its potential for *scale* and *depth*, i.e. the number of campaigns that can be run in parallel and the lengths of evolutionary ‘walks’ on the fitness landscape of the target gene [22]. In contrast, continuous DE makes mutations *in vivo*, specifically to the target gene, and couples the function of the target to growth of the platform cells. The target’s activity can then be improved simply by selecting for growth rate [8]. This procedure needs relatively little labor and so enables evolution campaigns of greater scale and depth than classical DE [22]. If the target enzyme’s function can be coupled to growth, continuous DE is thus a good option. In many cases, this coupling requires engineering the platform cells to make their growth depend on the target enzyme’s activity. A simple case is deleting an essential gene whose function can then be replaced by the target enzyme [23]. A further advantage of continuous over classical DE is that it can more readily cross fitness valleys between fitness peaks (Fig. 1), allowing broad exploration of the fitness landscape instead of being locked into stepwise ascent of the nearest peak even if that peak is not optimal [8, 23]. Lastly, when selecting the platform for continuous DE of a plant enzyme, note that yeast is generally preferable to *E. coli* because, as a eukaryote, it has redox, protein-folding, and proteolysis systems close to those in plants.

## Target selection

Here and in later sections, we start with general principles, then illustrate these using strawberry PAL.

### Principles

Selecting the target enzyme gene requires several types of information (Fig. 1, left). First, the enzyme must be known, or strongly inferred, to affect a trait of value to the end-user breeding program. Logically connecting enzyme and trait depends on biochemical and physiological knowledge. Second, a sequenced genome is necessary to pinpoint the sites to be edited and to predict potential off-target effects. Third, if a family of genes encodes the enzyme, a transcriptome is needed to identify which gene(s) are specifically expressed in the organ of interest at the appropriate stage (e.g. ripening fruit), and hence which to edit. Fourth, for continuous DE, it is essential to choose an enzyme whose activity can be robustly coupled to growth of a suitable platform strain, as said above.

**Figure 3:**
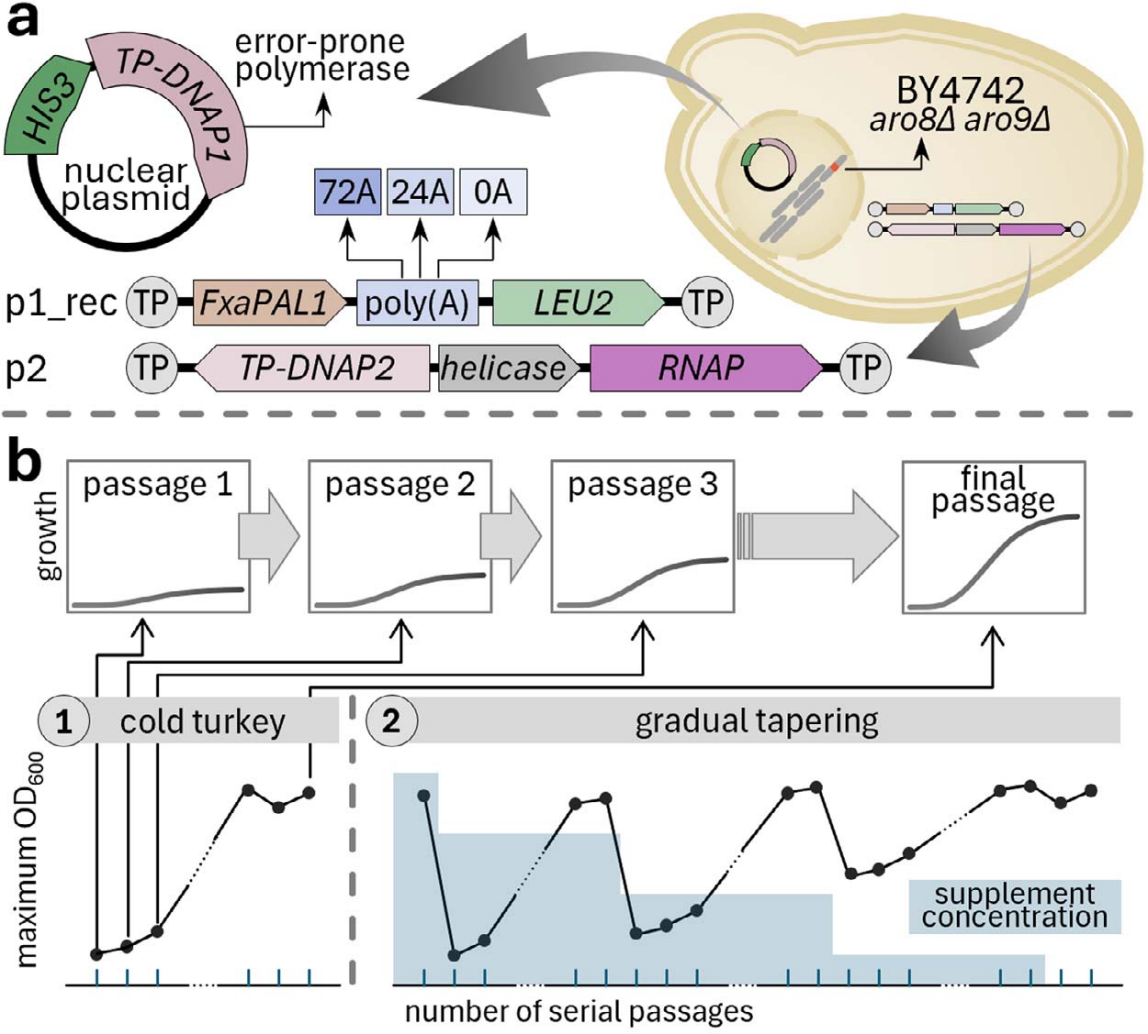
The yeast OrthoRep system for continuous DE and two of the selection strategies that can be used. (a) OrthoRep components. The target enzyme gene (GOI) is a cDNA recoded for expression in yeast. (b) Selection schemes for an enzyme on whose activity growth depends: 1, Cold turkey, where cells rely totally from the start on the activity of the target enzyme; and 2, Tapering, where the medium is supplemented with gradually decreasing levels of the metabolite that the target enzyme produces until the enzyme is improved enough to allow supplementation to stop.

### Illustration

Fruit color and firmness/skin toughness are desired traits in strawberries; they are respectively underlain by the levels of anthocyanins and lignin, which are products of the phenylpropanoid pathway [24, 25]. Increasing flux in this pathway can thus potentially increase anthocyanin and lignin synthesis [26–28]. In strawberry, as in all eudicots, the phenylpropanoid pathway proceeds from phenylalanine to *p*-coumaroyl-CoA via the sequential action of PAL, cinnamate 4-hydroxylase (C4H), and 4-coumarate-CoA ligase (4CL) (Fig. 2a). PAL coupling with C4H forms a rate-limiting step [29]. Further, the whole pathway is energetically wasteful because the *p*-hydroxy group on the ring is first removed from arogenate to give phenylalanine, then added back by C4H [30]. These constraints are avoided by converting tyrosine to *p*-coumarate via a tyrosine ammonia-lyase (TAL) (Fig. 2a). This efficient shortcut pathway occurs in bacteria [31] and grasses, in which it carries substantial flux [26, 32]. Grass TAL activity comes from a modified PAL (termed a PTAL) that has both PAL and TAL activities [26, 32]. Converting a monofunctional PAL into a bifunctional PTAL via GE is thus a strategy to increase flux to phenylpropanoids [26]. The tyrosine supply in strawberries appears adequate to support this [33, 34], and flux to arogenate via the shikimate pathway and later steps is feedback-regulated by both tyrosine and phenylalanine, so that the more of either that is consumed, the more is produced [35, 36]. Many whole-genome sequences of octoploid cultivated strawberry (*Fragaria × ananassa*) are available [37]; they contain seven PAL genes. Based on transcriptome data from 179 breeding lines, Fxa7Cg101819 (henceforth: FxaPAL1) has the highest expression in the fruit (Fig. 2b), suggesting a functional role in fruit ripening and softening. Moreover, its expression is essentially absent roots, leaves, and other tissues [38].

**Figure 4:**
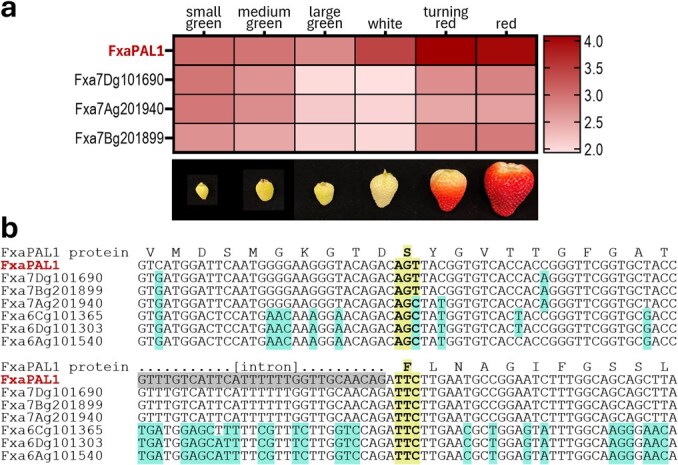
(a) Expression of FxaPAL1 and its three closest homoeologs during fruit ripening. Expression levels were normalized to *z-*scores with log_2_ (TPM + 1) for log-scale and represented as a heatmap. (b) DNA sequence alignment of FxaPAL1 and the 6 homoeologous genes in the regions of the S109 and F137 codons, which are highlighted. Differences from FxaPAL1 are highlighted in aqua. An intron is highlighted in gray.

## Setting up DE campaigns

### Principles

DE campaigns often simply start with a wildtype gene (recoded to optimize expression in the platform microbe) but, if there is enough knowledge about the enzyme, from the crop of interest or from another species, it is better to first make beneficial mutations based on this knowledge, i.e. to kick-start DE by leveraging rational design [20, 39]. Beneficial mutations can be predicted using protein sequence, 3D structure data, and information on the catalytic mechanism, and confirmed or disconfirmed by *in vitro* assays of recombinant enzymes.

### Illustration

Extensive information on the sequences, structures, and functions of PAL, TAL, and PTAL enzymes [26, 31, 40] enables prediction of several mutations likely to give FxaPAL1 physiologically significant TAL activity as a starting point for DE. The predicted mutations include A118G, S109I, and F137H. All three distinguish grass PTALs from PALs (Fig. S1); S109I and F137H have been shown experimentally to confer substantial TAL activity, particularly when combined [40], and A118 lies in the same flexible loop as S109 but nearer the active center (Fig. S2). All three mutations were made singly and in combination, and the resulting mutant enzymes and wildtype FxaPAL1 were expressed in *E. coli* and assayed for TAL and PAL activities (Fig. 2c and Table S1). The A118G mutation was not beneficial alone or when combined with F137H or S109I plus F137H. As predicted, the S109I–F137H double mutant had substantial TAL activity; its *k*_cat_ value of 0.02 s^−1^ and catalytic efficiency (*k*_cat_/*K*_M_) of 0.71 s^−1^ mM^−1^ with tyrosine as substrate are only about 5- to 10-fold less than those of natural PTALs [32, 40]. This double mutant is thus a suitable starting point for DE.

## Running DE campaigns

### Principles

As classical DE approaches for enzymes are well documented (e.g. [41, 42]), we focus here on the newer and more powerful continuous DE, specifically on the yeast OrthoRep system [9], which makes no off-target mutations and is particularly suitable for improving enzymes for plants [9, 23]. As said above, yeast and plants are both eukaryotic, and their cytoplasmic environments have similar cofactor concentrations, protein folding mechanisms, pH, and other features, making OrthoRep a suitable platform for evolving plant enzymes [23]. In OrthoRep (Fig. 3a), the target gene is expressed from p1, a linear cytoplasmic plasmid that is replicated by an error-prone, p1-specific DNA polymerase borne on a nuclear plasmid. The polymerase mutates the target at up to 10^6^ × the genomic mutation rate and can make almost as many transversion mutations as transitions, enabling a full range of amino acid changes [18]. A second cytoplasmic plasmid, p2, encodes an RNA polymerase that transcribes genes on p1 and p2. Target gene expression is tunable [43], and is coupled to growth as outlined above. Selection for growth rate can then be applied via various schemes (Fig. 3b) [8, 23].

### Illustration

The TAL activity of FxaPAL1 can be coupled to growth via a general strategy for enzymes that produce NH_3_ [44], which TAL does (Fig. 2a). Wildtype yeast cells can use NH_3_ or tyrosine as sole nitrogen source, but if the aromatic aminotransferase genes *ARO8* and *ARO9* are deleted the cells cannot use tyrosine [45]. When no NH_3_ or other nitrogen source is supplied in the medium, the ability of the target FxaPAL1 enzyme to produce NH_3_ from supplied tyrosine thus becomes limiting for growth [44]. OrthoRep campaigns in an *ARO8/9* deletant strain can then be run with the FxaPAL1 S109I–F137H mutant using an appropriate selection scheme. Estimates based on the *k*_cat_ value of the S109I–F137H mutant indicate that it would at first have too little TAL activity to support even slow growth (a doubling time of 24 h) with tyrosine as sole nitrogen source (Table S2). Supplementing tyrosine with NH_3_ at progressively lower levels (Fig. 3b, tapering strategy) is thus appropriate. As natural TAL enzymes have *k*_cat_ values up to several 1000-fold that of the S109I–F137H mutant [31, 46] it is reasonable to expect further mutations to improve TAL activity enough to contribute much or all of the nitrogen needed for growth (Table S2). When improved TAL activity (evidenced by faster growth during DE) is obtained, the mutations responsible are identified by sequencing individual clones or bulk populations [23, 47]. The effects of these mutations on TAL activity can then be confirmed and ranked by expressing the recombinant enzymes in *E. coli* and assaying activity *in vitro* as above. The set of mutations that gives the greatest improvement in catalytic efficiency is the output of the DE phase of the DE–GE pipeline (Fig. 1).

## The DE ➔ GE hand-off

### Principles

The principles are simply to use CRISPR/Cas technology to introduce the set of mutations, i.e. precise multinucleotide substitutions, prescribed by DE, into the target gene – and *only* that gene. Base editing and prime editing can both make such substitutions, but base editors only mediate transitions (C➔T or A➔G), which limits the amino acid sequence changes that they can accomplish [1, 2].

### Illustration

The mutations in FxaPAL1 will be introduced directly into an elite cultivar using protoplast transformation and regeneration methods, which significantly reduces the time required for extensive backcrossing [3]. To address the genomic complexity of octoploid strawberry, preliminary validation will be conducted using protoplast assays and transient expression in fruit tissues [3]. These assays will serve to test the efficiency of guide RNA (sgRNA) design and CRISPR vector construction, as well as to provide early insights into the potential phenotypic effects of expressing the edited FxaPAL1 in plant tissues. We take as examples the S109I and F137H mutations (Fig. 2c); DE is expected to add others (see above). The F137H mutation requires two transitions (TTC➔CAC) but the S109I mutation requires a transversion (AGT➔ATT), making prime editing the only option. Prime editing in strawberry is complicated by the multiple homeologous gene copies (Fig. 2b), so care is needed to target only FxaPAL1. The sequence similarity of the FxaPAL1 gene to its six homoeologs is critical to designing prime editing guide RNAs (pegRNAs) targeting the S109I and F137H mutations. At the DNA level, the three homoeologs on chromosome 6 are only ~75% identical to FxaPAL1, whereas the three on chromosome 7 are ~95% identical. FxaPAL1 is basically the only one of these chromosome 7 homoeologs expressed in ripening fruit (Fig. 4a). The sequence diversity in the regions flanking the S109 and F137 codons (Fig. 4b) indicates that gene-specific editing is feasible, e.g. using the prime editing vector pH-ePPE [48], engineered with strawberry U3 and Ubi [49, 50] promoters to drive pegRNA expression. pegRNAs targeting the specific sites for S109I and F137H mutations can be designed to be subgenome-specific or to mutate all copies in the four homoeologs on chromosome 7. The pegRNAs are unlikely to target the three homoeologs on chromosome 6 as there are too many sequence differences. For the S109I mutation, pegRNAs can be designed with homology arms (15–20 bp upstream and 10–15 bp downstream of the target site) complementary to the target site to ensure that the repair is directed correctly. An upstream SNP (G) and a downstream SNP (A) flanking the target site allow design of subgenome-specific edits for FxaPAL1 (Fig. 4b). As this is not possible for the F137H mutation, all four copies on chromosome 7 can be targeted and the mutated FxaPAL1 can be selected in selfed T1 populations [3]. Lines carrying the desired edits (i.e. S109I and F137H) can then be evaluated for overall plant phenotype and for fruit anthocyanin content and distribution, firmness, and skin toughness [3, 51–53].

## From GE to breeding

### Principles

Novel DE–GE alleles associated with desired characteristics enter the breeding program in much the same way as natural alleles, i.e. by crossing into elite breeding germplasm.

### Illustration

Edited FxaPAL1 lines showing desirable characteristics can be crossed as males with elite breeding parents to begin segregating out the transgene and simultaneously transmit the desired edits to additional genetic backgrounds. If the transgene is heterozygous, a single cross with a non-transgenic plant is typically sufficient to obtain transgene-free progeny. If the transgene is homozygous, two generations of crossing are usually required to segregate out the transgene. The presence of desired edits can be tracked by high-resolution melting (HRM) analysis and amplicon sequencing. Pollen can be harvested from single greenhouse plants of confirmed lines and applied to emasculated flowers of elite females in a greenhouse with pollinator exclusion. As edits are expected to be in the homozygous state, the progeny from crosses with other elite varieties will be heterozygous for the desired edits. These edits and their dosage can subsequently be screened via HRM markers and additional crosses made to obtain homozygous edits.

Note that implementation of this pipeline and the use of edited varieties would be guided by the specific regulations of the country in which it is applied [54]. The regulatory bypass for small edits (up to 3–4 bp changes) is based on the nature of the edit itself, not the genetic background. This means that if the edit is precise and does not introduce foreign DNA, it is less likely to be influenced by background-specific regulatory differences. In fact, this approach offers a more precise and targeted method for trait improvement, which could contribute to greater public acceptance of genome modification in plants. One of the key advantages of using DE is its ability to generate genetic variability in a specific target gene, further supporting this acceptance. Further, in our case, converting a PAL into a PTAL is a modest change, bioinspired by variability found in grasses but no other plants such as strawberry. There is thus minimal risk of introducing unwanted traits into the germplasm.

### Natural occurrence?

It is worth making a final point: Once beneficial mutations have been identified, it is prudent to check whether any of them occur naturally in the available germplasm. If so, this can save the time and expense of using GE to create these mutations.

## Afterword

This article sets out a roadmap for combining the power of DE with that of GE in practical breeding of specialty crops. We have used a PAL gene implicated in strawberry fruit quality as an example, but the DE–GE pipeline is a general one that can in principle be applied to any enzyme-controlled trait. The rationale for a roadmap is this: While much has been written about the potential of DE in agriculture, with the exception of herbicide resistance (e.g. [55, 56]), this literature consists more of imaginative conceptual visions than of concrete plans for implementation (e.g. [14, 57–60]). Much has also been written about using technologies such as machine learning and high-throughput screening to enhance the rational design element of DE by predicting and screening improved plant enzyme variants (e.g. [13, 14]). Implementing these ideas likewise lies mainly in the future. It is now time to move beyond visions to realistic programs to improve metabolic traits important to consumers and producers [61, 62].

## Supplementary Material

Web_Material_uhaf203

## Data Availability

Not applicable as no datasets were generated or analyzed during this study beyond those reported in the text or supplementary data.
