# Supplementary material for: Building a directed evolution–genome editing pipeline for metabolic traits in specialty crop breeding
Source: Hortic Res. 2025 Oct 25;12(11):uhaf203. doi: 10.1093/hr/uhaf203 (PMC12574542; doi:10.1093/hr/uhaf203)
Supplement: Web_Material_uhaf203 [file web_material_uhaf203.zip › Figure S1.pdf]

Fig. S1. Multiple sequence alignment FxaPAL1 with PALs and PTALs from grasses and the close grass relative *Joinvillea ascendens*.

Residues that fully differentiate PALs and PTALs are marked *. For residues shown to be required for PTAL activity in *J. ascendens* and Arabidopsis, the asterisk is underscored. The MIO (3,5-dihydro-5-methylidene-4H-imidazol- 4-one) cofactor precursor tripeptide residues are marked +++.

```
                                                                                                                                    *
FxaPAL1              MALNGNGNC NGAAAVGSFCQGGGLLHDPLNWNLAAEGLKGSHLDELKRMVNDYRKPVVMLGGESLTVGQVAAIANHDG  GVRVELSEEKRAGVKASSDWVMDSMGKGTDSYGV
AtPAL1    MEINGA  HKSNGGGVDAMLCGGDIKTKNMVI  NAE DPLNWGAAAEQMKGSHLDEVKRMVAEFRKPVVNLGGETLTIGQVAAISTIGN  SVKVELSETARAGVNASSDWVMESMNKGTDSYGV
JaPAL            MECENGNVAAVAAVNGGNGLCLQKP  QHA DPLNWGKAAGELMGSHLEEVKRMVAEFRAPVVKIEGASLRIAQVAAVAAGEAA AAKVELDESSRGRVKASSDWVMSSMMNGTDSYGV
ZmPAL7           MECENGRGV AATNSDSLCMAT     PRA DPLNWGKAAEELMGSHLDEVKRMVAEYRQPLVKIEGASLSIAQVAAVATGAG  EARVELDESARSRVKASSDWVMTSMMNGTDSYGV
ZmPAL4           MECDNGR V AATNGDSLCMAL     PRAADPLNWGKAAEEMMGSHLDEVKRMVAEYRQPLVKIEGASLRIAQVAAVAAGAG  EARVELDESARGRVKASSDWVRDSMMNGTDSYGV
OsPAL            MECENGR V SANGMSGLCVAA     PRA DPLNWGKATEEMTGSHLDEVKRMVAEYRQPLVKIEGASLRIAQVAAVAA AG  EARVELDESARERVKASSDWVMNSMMNGTDSYGV
BoPAL            MPREDGH V AANG NGLCMAA     PRA DPLNWGKAAEELMGSHLDEVKRMVAEYRQPVVKIEGASLRIAQVAAVAV AG  DAKVELDESARERVKASSDWVMNSMMNGTDSYGV
BdPAL            MECENGQ F AANG TGLCMAT     PRA DPLNWGKAAEELTGSHLDEVKRMVAEYRKPVVTIEGATLSIAKVAAVAA AG  EAKVELDESARERVKASSDWVMNSMANGVDSYGV
ZmPAL1           MESEAGLLVRSSLNGEGLCMPA     PRA DPLNWGKAAEGLSGSHLDEVKRMVAEFRDPLVKIQGASLSVAQVAAVAVGAGGGEARVELDESARERVRASSDWVMGSMMNGTDSYGV
ZmPAL2           MECETGLV   RSLNGEGLCMSAAAAAPRA DALNWGKAAEDLSGSHLDEVKRMVVEFREPLVRIQGASLSIGQVAAVAAGAGG EARVELDESARGRVKASSDWVMSSMMNGTDSYGV
ZmPAL3           MECENGHL  AASGNGGVCLATPA   PRA DPLNWAKAAEDLAGSHLDAVKRMVEEYRRPLVKIEGASLTVAQVAAVAA AG  EARVELDESARGRVKASSDWVMDSMMNGTDSYGV
JaPTAL                            MAFQNDNVLCIKK DPLNWGKAAEELTGSHLDEVKRMVAESRTPVVKIQGASLRIGQVAAVAV RETGVAKVELDESARGRVKASSDWIVNSVANGGDIYGV
ZmPTAL1                       MAGNGAIVES        DPLNWGAAAAELAGSHLDEVKRMVAQARQPVVKIEGSTLRVGQVAAVASAKDASGVAVELDEEARPRVKASSEWILDCIAHGGDIYGV
OsPTAL                        MAGNGPINKE        DPLNWGAAAAEMAGSHLDEVKRMVAQFREPLVKIQGATLRVGQVAAVAQAKDAAGVAVELDEEARPRVKASSEWILNCIAHGGDIYGV
BoPTAL                        MAGNGPIVKD        DPLNWGAAAAELTGSHFDEVKRMVAQFREPVIKIEGASLRVGQVAAVAQAKDVSGVAVELDEEARPRVKASSEWILNCLAHGGDIYGV
BdPTAL1                       MAGNGAISEK        DPLNWGAAAAELTGSHLDEVKRMVAQFREPVVKIEGASLRVGQVAAVAQAKDAAGVSVELDEEARPRVKASSEWILSCLAAGGDIYGV
ZmPTAL2                       MASNTAILES        DPLNWGKAAAELTGSHLDEVKRMVAQFRDPVVKIEGSSLRVGQVAAVAAAKDASGVAVELDEEARPRVKASSEWILDCIAHGGDIYGV
SoPTAL1                       MASNTAILES        DPLNWGKAAAELTGSHLDEVKRMVAQFRDPVVKIEGSTLRVGQVAAVAAAKDASGVAVELDEEARPRVKASSEWILDCIAHGGDIYGV
SoPTAL2                       MAGNGAIVES        DPLNWGAAAAELAGSHLDEVKRMVAQARQPVVKIEGSTLRVGQVAAVAAAKDASGVAVELDEEARPRVKASSEWILDCIAHGGDIYGV

               *                  *                                                                   +++
FxaPAL1   TTGFGATSHRRTKNGGALQKELIRFLNAGIFGSSLDSTHKLPHTATRAAMLVRINTLLQGYSGIRFEILEAITKLLNGNITPCLPLRGTISSSGDLVPLSYIAGLLIGRPNSKSIGPNGETLTPA
AtPAL1    TTGFGATSHRRTKNGVALQKELIRFLNAGIFGSTKETSHTLPHSATRAAMLVRINTLLQGFSGIRFEILEAITSFLNNNITPSLPLRGTITASGDLVPLSYIAGLLTGRPNSKATGPNGEALTAE
JaPAL     TTGFGATSHRRTKEGGALQRELIRFLNAGVFGTGSD GHVLPAAATRAAMLVRINTLLQGYSGIRFEILEAITALLNAGVTPCLPLRGTITASGDLVPLSYIAGLITGRPNSVAVAPDGRKVDAA
ZmPAL7    TTGFGATSHRRTKEGGALQRELIRFLNAGAFGTGAD GHVLPAETTRAAMLVRINTLLQGYSGIRFEILEAIVKLLNANVTPCLPLRGTVTASGDLVPLSYIAGLVTGRENSVAVAPDGSKVNAA
ZmPAL4    TTGFGATSHRRTKEGGALQRELIRFLNAGAFGIGTDAGHVLPAEATRAAMLVRINTLLQGYSGIRFEILEAIVKLLNANVTPCLPLRGTVTASGDLVPLSYIAGLVTGRENAVAVAPDGTKVNAA
OsPAL     TTGFGATSHRRTKEGGALQRELIRFLNAGAFGTGTD GHVLPAEATRAAMLVRINTLLQGYSGIRFEILEAIAKLLNANVTPCLPLRGTITASGDLVPLSYIAGLVTGRENAVAVAPDGSKVNAA
BoPAL     TTGFGATSHRRTKEGGALQRELIRFLNAGAFGTGCD GHVLPAEATRAAMLVRINTLLQGYSGIRFEILEAITKLLNANVTPCLPLRGTVTASGDLVPLSYIAGLVTGRENSVAVAPDGRKVNAA
BdPAL     TTGFGATSHRRTKEGGALQRELIRFLNAGAFGTGSD GHVLPAGATRAAMLVRINTLLQGYSGIRFEILEAIAKLLNANVTPCLPLRGTITASGDLVPLSYIAGLVTGRENSVAVTPDGRKVNAA
ZmPAL1    TTGFGATSHRRTKEGGALQRELIRFLNAGAFGTGAD GHVLPAEATRAAMLVRVNTLLQGYSGIRFEILEAIAKLLNANVTPCLPLRGTITASGDLVPLSYIAGLITGRQNSVAVDPDGRKVGAA
ZmPAL2    TTGFGATSHRRTKEGGALQRELIRFLNAGAFGTGAD GHVLPAEATRAAMLVRINTLLQGYSGIRFEILEAIAKLLNANVTPCLPLRGTITASGDLVPLSYIAGLITGRQNSVAVGPDGRKVGAA
ZmPAL3    TTGFGATSHRRTKEGGALQRELIRFLNAGAFGTGDD GHVLPAAATRAAMLVRINTLLQGYSGIRFEILEAIAALLNTNVTPCLPLRGTITASGDLVPLSYIAGLVTGRPNSTAVAPDGRKVGAA
JaPTAL    TTGFGGTSHRRTRDGPALQIELLRHLNAGIFGTGTD GHVLPSAVTRAAMFVRINTLLQGYSGIRFEILEAIAALLNAGVTPCLPLRGTITASGDLIPLSYIAGLITGRPNAVAIAPDGRKVDAA
ZmPTAL1   TTGFGGTSHRRTKDGPALQVELLRHLNAGIFGTGSD GHTLPSEVTRAAMLVRINTLLQGYSGIRFEILEAITKLLNTGVSPCLPLRGTITASGDLVPLSYIAGLITGRPNAQAVTVDGRKVDAA
OsPTAL    TTGFGGTSHRRTKDGPALQVELLRHLNAGIFGTGSD GHTLPSETVRAAMLVRINTLLQGYSGIRFEILEAITKLLNTGVTPCLPLRGTITASGDLVPLSYIAGLITGRPNAQAISPDGRKVDAA
BoPTAL    TTGFGGTSHRRTKDGPALQVELLRHLNAGIFGTGTD GHTLPSEVTRAAMLVRINTLLQGYSGIRFEILEAITKLINTGVTPCLPLRGTITASGDLVPLSYIAGLITGRPNAQAVAPDGRKVDAA
BdPTAL1   TTGFGGTSHRRTKDGPALQVELLRHLNAGIFGTGSD GHSLPAEVTRAAMLVRINTLLQGYSGIRFEILEAITKLLNTGVSPCLPLRGTITASGDLVPLSYIAGLITGRPNAQATTADGRKVDAA
ZmPTAL2   TTGFGGTSHRRTKDGAALQVELLRHLNAGIFGNGSD GHTLPSEVSRAAMLVRINTLLQGYSGIRFEILEAITKLINTGVSPCLPLRGTITASGDLVPLSYIAGLITGRPNAQAVTVDGRKVDAA
SoPTAL1   TTGFGGTSHRRTKDGPALQVELLRHLNAGIFGNGSD GHTLPSEVSRAAMLVRINTLLQGYSGIRFEILEAITKLLNTGVSPCLPLRGTITASGDLVPLSYIAGLITGRPNAQAVTVDGRKVDAA
SoPTAL2   TTGFGGTSHRRTKDGPALQVELLRHLNAGIFGTGTD GHTLPSEVSRAAMLVRINTLLQGYSGIRFEILEDITKLLNTGVSPCLPLRGTITASGDLVPLSYIAGLITGRPNAQAVTVDGRKVDAA

                                     *
FxaPAL1   EAFKLAGIEGDFFELQPKEGLALVNGTAVGSGMASVVLFDANTLAVLSEIMSAIFAEVMQGKPEFTDHLTHKLKHHPGQIEAAAIMEHILDGSSYVKEAKKVHEMDPLQKPKQDRYALRTSPQWL
AtPAL1    EAFKLAGISSGFFDLQPKEGLALVNGTAVGSGMASMVLFETNVLSVLAEILSAVFAEVMSGKPEFTDHLTHRLKHHPGQIEAAAIMEHILDGSSYMKLAQKLHEMDPLQKPKQDRYALRTSPQWL
JaPAL     EAFKIAGIQHGFFELQPKEGLAMVNGTAVGSGLASTVLFEANILTILAEVLSAVFCEVMTGKPEYTDHLTHKLKHHPGQIEAAAIMEHILEGSSYMKLAKKLGDLDPLMKPKQDRYALRTSPQWL
ZmPAL7    EAFKIAGIQGGFFELQPKEGLAMVNGTAVGSGLASTVLFEANILAILAEVLSAVFCEVMNGKPEYTDHLTHKLKHHPGQIEAAAIMEHILEGSSYMKLAKKLGELDPLMKPKQDRYALRTSPQWL
ZmPAL4    EAFRIADIQSGFFELQPKEGLAMVNGTAVGSGLASTVLFEANVLAVLAEVLSAVFCEVMNGKPEYTDHLTHKLKHHPGQIEAAAIMEHILEGSSYMKLAKKLGELDPLMKPKQDRYALRTSPQWL
OsPAL     EAFKIAGIQGGFFELQPKEGLAMVNGTAVGSGLASTVLFEANILAILAEVLSAVFCEVMNGKPEYTDHLTHKLKHHPGQIEAAAIMEHILEGSSYMKHAKKLGELDPLMKPKQDRYALRTSPQWL
BoPAL     EAFKIAGIQGGFFELQPKEGLAMVNGTAVGSGLASTVLFEANILAILAEVLSAVFCEVMNGKPEYTDHLTHKLKHHPGQIEAAAIMEHILEGSSYMKLAKKLGDLDPLMKPKQDRYALRTSPQWL
BdPAL     EAFKIAGIHGGFFELQPKEGLAMVNGTAVGSGLASTVLFEANILAVLAEVISAVFCEVMNGKPEFTDHLTHKLKHHPGQIEAAAIMEHILEGSSYMKLAKKLGDLDPLMKPKQDRYALRTSPQWL
ZmPAL1    EAFKIAGIEHGFFELQPKEGLAMVNGTAVGSGLASTVLFEANVLAVLAEVISAVFCEVMTGKPEFTDHLTHKLKHHPGQIEAAAVMEHILEGSSYMKLAKRLGELDPLMKPKQDRYALRTSPQWL
ZmPAL2    EAFRIAGIEHGFFELQPKEGLAMVNGTAVGSGLASTVLFEANVLAVMAEVISAVFCEVMTGKPEFTDHLTHKLKHHPGQIEAAAIMEHVLEGSSYMKLAKKLGELDPLMKPKQDRYALRTSPQWL
ZmPAL3    EAFEIAGIQHGFFELQPKEGLAMVNGTAVGSGLASMVLFEANVLAVLAEVMSAVFCEVMNGKPEYTDHLTHKLKHHPGQIESAAIMEHILEGSSYMALAKKLGELDPLMKPKQDRYALRTSPQWL
JaPTAL    EAFKIAGIPHGFFKLQPKEGLAIVNGTSVGAALASTVLFDANILAILAEVLSAVFCEVMTGKPEYTDHLTHRLKHHPGQIEAAAIMEHILEGSSFMKLAKKLGELDPLLKPKQDRYALRTAPQWL
ZmPTAL1   EAFKIAGIEGGFFKLNPKEGLAIVNGTSVGSALAATVMYDANVLAVLSEVLSAVFCEVMNGKPEYTDHLTHKLKHHPGSIEAAAIMEHILDGSSFMKQAKKLNELDPLLKPKQDRYALRTSPQWL
OsPTAL    EAFKLAGIEGGFFTLNPKEGLAIVNGTSVGSALAATVMFDANILAVLSEVLSAVFCEVMNGKPEYTDHLTHKLKHHPGSIEAAAIMEHILAGSSFMSHAKKVNEMDPLLKPKQDRYALRTSPQWL
BoPTAL    EAFKIAGIEGGFFKLNPKEGLAIVNGTSVGSALAATVLYDCNVLAVLSEVLSAVFCEVMNGKPEYTDHLTHKLKHHPGSIEAAAIMEHILAGSSFMSHAKKVNEMDPLLKPKQDRYALRTSPQWL
BdPTAL1   EAFKVAGIEGGFFTLNPKEGLAIVNGTSVGSALAATVLFDCNVLAVLSEVLSAVFCEVMNGKPEFTDHLTHKLKHHPGSIEAAAIMEHILAGSSFMSHAKKVNEIDPQLKPKQDRYALRTSPQWL
ZmPTAL2   EAFKAAGIEGGFFKLNPKEGLAIVNGTSVGSALAAMVCFDANVLAVLSEVLSAVFCEVMNGKPEYTDHLTHKLKHHPGSIEAAAIMEHILDGSSFMKHAKEVNAMDPLLKPKQDRYALRTSPQWL
SoPTAL1   EAFKVAGIEGGFFKLNPKEGLAIVNGTSVGSALAAMVCFDANVLAVLSSVLSAVFCEVMNGKPEYTDHLTHKLKHHPGSIESAAIMEHILDGSSFMKHAKEVNAMDPLLKPKQDRYALRTSPQWL
SoPTAL2   EAFKVAGIEGGFFKLNPKEGLAIVNGTSVGSPLAAMVCFDANVLAVLSSVLSAVFCEVMNGKPEYTDHLTHKLKHHPGSIESAAIMEHILDGSSFMKHAKEVNAMDPLLKPKQDRYALRTSPQWL
```

```
                                                                                  *
FxaPAL1   GPLIEVIRSSTKMIEREINSVNDNPLIDVSRNKALHGGNFQGTPIGTAMDNTRLALASIGKLIFAQFSELVNDFYNNGLPSNLSGGSNPSLDYGFKGAEIAMASYCSELQFLANPVTNHVQSAEQ
AtPAL1    GPQIEVIRYATKSIEREINSVNDNPLIDVSRNKAIHGGNFQGTPIGVSMDNTRLAIAAIGKLMFAQFSELVNDFYNNGLPSNLTASRNPSLDYGFKGAEIAMASYCSELQYLANPVTSHVQSAEQ
JaPAL     GPQIEVIRASTKSIEREINSVNDNPLIDVSRGKALHGGNFQGTPIGVSMDNTRLAIAAIGKLMFAQFSELVNDFYNNGLPSNLSGGRNPSLDYGFKGAEIAMASYCSELQFLANPVTNHVQSAEQ
ZmPAL7    GPQIEVIRAATKSIEREINSVNDNPLIDVARSKALHGGNFQGTPIGVSMDNTRLAIAAIGKLMFAQFSELVNDYYNNGLPSNLSGGRNPSLDYGFKGAEIAMASYCSELQFLGNPVTNHVQSAEQ
ZmPAL4    GPQIEVIRASTKSIEREINSVNDNPLIDVARSKALHGGNFQGTPIGVSMDNTRLAVAAIGKLMFAQFSELVNDYYNNGLPSNLSGGRNPSLDYGFKGAEIAMASYCSELQFLGNPVTNHVQSAEQ
OsPAL     GPQIEVIRAATKSIEREINSVNDNPLIDVSRGKALHGGNFQGTPIGVSMDNTRLAIAAIGKLMFAQFSELVNDFYNNGLPSNLSGGRNPSLDYGFKGAEIAMASYCSELQFLGNPVTNHVQSAEQ
BoPAL     GPQIEVIRAATKSIEREINSVNDNPLIDVSRNKALHGGNFQGTPIGVSMDNTRLAIAAIGKLMFAQFSELVNDFYNNGLPSNLSGGRNPSLDYGFKGAEIAMASYCSELQFLGNPVTNHVQSAEQ
BdPAL     GPQIEVIRAATKSIEREINSVNDNPLIDVSRGKAIHGGNFQGTPIGVSMDNTRLALAAIGKLMFAQFSELVNDFYNNGLPSNLSGSRNPSLDYGFKGAEIAMASYCSELQFLGNPVTNHVQSAEQ
ZmPAL1    GPQIEVIRFATKSIEREINSVNDNPLIDVSRGKALHGGNFQGTPIGVSMDNTRLALAAIGKLMFAQFSELVNDYYNNGLPSNLSGGRNPSLDYGFKGAEIAMASYCSELQFLGNPVTNHVQSAEQ
ZmPAL2    GPQIEVIRFATKSIEREINSVNDNPLIDVSRGKALHGGNFQGTPIGVSMDNTRLALAAIGKLMFAQFSELVNDYYNNGLPSNLSGGRNPSLDYGFKGAEIAMASYCSELQFLGNPVTNHVQSAEQ
ZmPAL3    GPQIEVIRSATKSIEREINSVNDNPLIDVSRGKALHGGNFQGTPIGVSMDNTRLAVAAIGKLMFAQFSELVNDFYNNGLPSNLSGGRNPSLDYGFKGAEIAMASYCSELQFLANPVTNHVQSAEQ
JaPTAL    GPQIEVIRAATKSIEREINSVNDNPIIDVSRGKALHGGNFQGTPIGVSMDNTRLAIANIGKLMFAQFSELVNDFYNNGLTSNLAGGRNPSLDYGFKGTEIAMASYCSELQFLANPVTNHVQSAEQ
ZmPTAL1   GPQIEVIRAATKSIEREVNSVNDNPVIDVHRGKALHGGNFQGTPIGVSMDNARLAIANIGKLMFAQFSELVNEFYNNGLTSNLAGSRNPSLDYGFKGTEIAMASYCSELQYLGNPITNHVQSAEQ
OsPTAL    GPQIEVIRAATKSIEREVNSVNDNPVIDVHRGKALHGGNFQGTPIGVSMDNARLAIANIGKLMFAQFSELVNEFYNNGLTSNLAGSRNPSLDYGFKGTEIAMASYCSELQYLANPITNHVQSAEQ
BoPTAL    GPQIEVIRAATKSIEREVNSVNDNPVIDVHRGKALHGGNFQGTPIGVSMDNTRLAIANIGKLMFAQFSELVNEFYNNGLTSNLAGSRNPSLDYGFKGTEIAMASYCSELQYLANPITNHVQSAEQ
BdPTAL1   GPQIEVIRSATKSIEREVNSVNDNPVIDVHRGKALHGGNFQGTPIGVSMDNTRLAIANIGKLMFAQFSELVNEFYNNGLTSNLAGSRNPSLDYGFKGTEIAMASYCSELQYLANPVTNHVQSAEQ
ZmPTAL2   GPQIEVIRAATKSIEREVNSVNDNPVIDVHRGKALHGGNFQGTPIGVSMDNARLAVANIGKLMFAQFSELVNEFYNNGLTSNLAGSRNPSLDYGFKGTEIAMASYCSELQYLANPITNHVQSAEQ
SoPTAL1   GPQIEVIRAATKSIEREVNSVNDNPVIDVHRGKALHGGNFQGTPIGVSMDNARLAIANIGKLMFAQFSELVNEFYNNGLTSNLAGSRNPSLDYGFKGTEIAMASYCSELQYLANPITNHVQSAEQ
SoPTAL2   GPQIEVIRAATKSIEREVNSVNDNPVIDVHRGKALHGGNFQGTPIGVSMDNARLAIANIGKLMFAQFSELVNEFYNNGLTSNLAGSRNPSLDYGFKGTEIAMASYCSELQYLANPITNHVQSAEQ

                     *
FxaPAL1   HNQDVNSLGLISSRKTSEAVDILKLMSSTFLVALCQAVDLRHMEENLKSVVKNTVSKVARRTLTVALNGELHPSRFSEKHLLSVVDREYLFSYIDDPCLATYPLMQKLRAELVEHALNNGDKEK
AtPAL1    HNQDVNSLGLISSRKTSEAVDILKLMSTTFLVAICQAVDLRHLEENLRQTVKNTVSQVAKKVLTTGVNGELHPSRFCEKDLLKVVDREQVYTYADDPCSATYPLIQKLRQVIVDHALINGESEK
JaPAL     HNQDVNSLGLISSRKTAEAVDILKLMSSTFLIALCQAIDLRHLEENLKSAVKNCVAQVAKKALTLNTVGDLHNARFSEKDLLTAIDREALFAYADDPCNPNYPLMQKLRAVLVEHALANGEAEH
ZmPAL7    HNQDVNSLGLISSRKTAEAIEILKLMSSTFLIALCQAVDLRHIEENVKSAVKSCVMTVAKKTLSTNSTGGLHVARFCEKDLLQEIEREAVFAYADDPCSANYPLMKKLRNVLVERALANGAAEF
ZmPAL4    HNQDVNSLGLISSRKTAEAIEILKLMSSTFLIALCQAVDLRHIEENVKSAVKSCVMTVAKKTLSTNSTGGLHVARFCEKDLLQEIEREAVFAYADDPCSANYPLMKKLRNVLVERALANGTAEF
OsPAL     HNQDVNSLGLISSRKTDEAIDILKLMSSTFLIALCQAVDLRHIEENVKSAVKSCVMTVAKKTLSTNSTGDLHVARFCEKDLLKEIDREAVFAYADDPCSHNYPLMKKLRNVLVERALANGAAEF
BoPAL     HNQDVNSLGLISSRKTAEAIDILKIMSSTFLVALCQAIDLRHIEENVKSAVKSCVMTVAKKTPSTNSTGDLHVARFCEKDLLKEIDREAVFAYADDPCSPNYPLMKKLRSVLVESALANGMAEF
BdPAL     HNQDVNSLGLISSRKTAEAVEILKLMSSTFLVALCQAIDLRHIEENVKSAVKSCVMTVAKRTLSTNSTGGLHVARFCEKDLLQEIEREAVFAYADDPCSANYPLMKKLRGVLVERALSNGKAEF
ZmPAL1    HNQDVNSLGLISSRKTAEAIDILKLMSSTFLIALCQAIDLRHLEENVKAAVKNCVTQVAKKSLSLNARGGLHNARFCEKDLQTAIDREAVFAYADDPCSPNYALMQKLRAVLVEHALANGDAER
ZmPAL2    HNQDVNSLGLISSRKTAEAVDILKLMTSTFLIALCQAIDLRHLEENVKAAVKNCVTQVAKKSLSLNARGGLHNARFCEKDLQTAIDREAVFAYADDPCSPNYPLMQKLRAVLVEHALANGDAER
ZmPAL3    HNQDVNSLGLISSRKTAEAVDVLKLMSSTFLVALCQAIDLRHLEENLRSAVKRCVTVVARKTLSTGATGALHDARFCEKDLLTAIEREAVFAYADDPCSATYPLMQKMRSVLVEHALANGEAER
JaPTAL    HNQDVNSLGLVSARKTAEAVDILKLMSSTFLIALCQAIDLRHLEENLKSAVKNCVAQVAKKVLTVNTVGDLHNARFSEKDLLTAIDREAVFTYADDPCSPNYPLMQKVRAVLVEHALANGEAER
ZmPTAL1   HNQDVNSLGLVSARKTAEAIDILKLMSSTYIVALCQAVDLRHLEENIKASVKNTVTQVAKKVLTMNPSGELSSARFSEKELISAIDREAVFTYAEDAASGSLPLMQKLRAVLVDHALSSGDA
OsPTAL    HNQDVNSLGLVSARKTLEAVDILKLMTSTYIVALCQAVDLRHLEENIKSSVKNCVTQVAKKVLTMNPTGDLSSARFSEKNLLTAIDREAVFSYADDPCSANYPLMQKLRAVLVEHALTSGDA
BoPTAL    HNQDVNSLGLVSARKTAEAVDILKLMSSTYMVALCQAVDLRHLEENIKSSVKNCVTQVAKKVLTMNPTGDLSSARFSEKNLLTAIDREAVFTYADDPCSANYPLMQKLRAVLVDHALTSGDA
BdPTAL1   HNQDVNSLGLVSARKTAEAVDILKLMSSTYMVALCQAVDLRHLEENIKASVKNCVTQVSKKVLTMNPTGDLSSARFSEKSLLTAIDREAVFSYADDACSANYPLMQKLRAVLVDHALTSSGVDNAG
ZmPTAL2   HNQDVNSLGLVSARKTAEAVDILKLMSSTYMVALCQAVDLRHLEENLKSAVKSCVMAVARKVLTTSLGGDLHSARFSEKALLTAIDREAVYGYYDDPCSANSPLMKKIRAVLVDHALASGEAEK
SoPTAL1   HNQDVNSLGLVSARKTAEAVDILKLMSSTYMVALCQAVDLRHLEENLKSAVKNSVMAVARKVLTTSLDGDLHSARFSEKALLTAIDREAVYGYYDDPCSANSPLMKKIRAVLVDHALANGEAEK
SoPTAL2   HNQDVNSLGLVSARKTAEAIDILKLMSSTYIVALCQAIDLRHLEENIKTSVKNTVTQVAKKVLTMNPSGDLSSARFSEKELITAIDREGVFTYAEDPASGSLPLMQKLRSVLVDHALSSGDA

FxaPAL1   STNTSIFLKIAAFEEELKSVLPKEVDNARMEIENGKAEIANRIKECRSYPLYRFVREELGTSLLTGEKIRSPGEECDKVFNAICAGKLIDPLLECLKEWNGAPLPIS
AtPAL1    NAVTSIFHKIGAFEEELKAVLPKEVEAARAAYDNGTSAIPNRIKECRSYPLYRFVREELGTELLTGEKVTSPGEEFDKVFTAICEGKIIDPMMECLNEWNGAPIPIC
JaPAL     VATTSVFAKITKFEEELRATLPKEVEAARVAVENGTAPTPNRIKECRSYPLYRFVREELGTEYLTGEKLRSPGEECNKVFVAINQGKLIDPLLECLKEWNGEPLPIC
ZmPAL7    NAETSVFAKVAQFEEDLRAALPKAVEAARAAVENGTAGIPNRIAECRSYPLYRFVREELGAVYLTGEKTRSPGEELNKVLVAINQGKHIDPLLECLKEWNGEPLPIC
ZmPAL4    DAETSVFAKVAQFEEELRTALPSAVEAARAAVENGTAAIPNRIAECRSYPLYRFVREELGAVYLTGEKTRSPGEELNKVLVAINQGKHIDPLLECLKEWNGEPLPIC
OsPAL     NADTSVFAKVAQFEEELRATLPGAIEAARAAVENGTAAIPSRITECRSYPLYRFVREELGTKYLTGEKTRSPGEELNKVLVAINEGKHIDPLLECLKEWNGEPLPIC
BoPAL     NAETSIFARVALFEEELRAALPRAVEAARASVENGTAAAPNRITECRSYPLYRFVREELGTEYLTGEKTRSPGEELNKVLLAINQGKHIDPLLECLKEWNGEPLPIC
BdPAL     NAETSVFAKVAQFEEELRTALPKAVEAARSAVESGTAATPNRIKECRSYPLYRFVREELGTAYLTGEKTRSPGEELNKVLVAINQGKHIDPLLECLKEWNGEPLPIC
ZmPAL1    DVDTSIFAKVAEFEQQVRAALPKEVEAARAAVENGSPLVPNRIKECRSYPLYRFVREEVGTKYLTGEKTRSPGEELNKVLVAINQRKHIDPLLECLKEWNGEPLPLC
ZmPAL2    AAETSIFAKVAEFEQQVRAALPKEVEAARAAVESGSPLVPNRIRECRSYPLYRFVREDVGTEYLTGERTRSPGEELNKVLVAINQRKHIDPLLECLKEWNGAPLPLC
ZmPAL3    DPDTSVFAKVATFEEELRAALPREVDAARAAVESGTAAIRNRIAECRSYPLYRFVREELGTEYLTGEKARSPGEEVDKVFVAMNLGKHIDAVLECLKEWNGEPLPIC
JaPTAL    VANTSVFAKITKFEEELRATLPKEVEAARVAVENRTAPTQNRIKESRSYPLYRFVREDLGTAYLTGEKLRSPGEECNKVFVAINQGKLIDPLLECLKKWNGEPLPIC
ZmPTAL1   EREPSVFSKITRFEEELRAVLPQEVEAARVAVAEGTAPVANRIADSRSFPLYRFVREELGCVFLTGERLKSPGEECNKVFVGISQGKLVDPMLECLKEWDGKPLPINVK
OsPTAL    EPEASVFSKITKFEEELRSALPREIEAARVAVANGTAPVANRIVESRSFPLYRFVREELGCVFLTGEKLKSPGEECNKVFLGISQGKLIDPMLDCLKEWNGEPLPIN
BoPTAL    EREPSVFSKITKFEEELRSALPREIEAARVAVADGTAPIANRIKESRSFPVYRFVREELGCVYLTGEKLKSPGEECNKVFIGISQGKLIDPMLECLKEWNGEPLPIN
BdPTAL1   ESEATVFSKINKFEEELRAALPREIEAARVAFEKGTAPIPNLIKDSRSFPLYRFVREELGCVYLTGEKLLSPGEECNKVFIGISQGKLIDPMLECLKEWNGEPLPINVV
ZmPTAL2   DASASVFSKINRFEEELREALPREMEAARVAFETGAAPIANRIKESRSYPLYRFIRQDLGAVYLTGEKLKSPGEECNKVFLALSEGKLIDPMLDCLKEWDGKPLPIC
SoPTAL1   DASASVFSKINRFEETLREVLPREMEAARVAFETGTAPIANRIKESRSYPLYRFIRQDLGAVYLTGEKLKSPGEECNKVFLALSEGKLIDPMLECLKEWDGKPLPIC
SoPTAL2   EREPSVFSKITRFEEELRAVLPREVEAARVAVAEGTAPVANRIADSRSFPLYRFVREELGCVFLTGEKLKSPGEECTKVFNGISQGKLVDPMLECLKEWDGKPLPINIVN
```

Species abbreviations: At, *Arabidopsis thaliana*; Ja, *Joinvillea ascendens*; Zm, *Zea mays*; Os, *Oryza sativa*; Bo, *Bambusa oldhamii*; Bd, *Brachypodium distachyon.*
