# Supplementary material for: Building a directed evolution–genome editing pipeline for metabolic traits in specialty crop breeding
Source: Hortic Res. 2025 Oct 25;12(11):uhaf203. doi: 10.1093/hr/uhaf203 (PMC12574542; doi:10.1093/hr/uhaf203)
Supplement: Web_Material_uhaf203 [file web_material_uhaf203.zip › Figure S2.pdf]

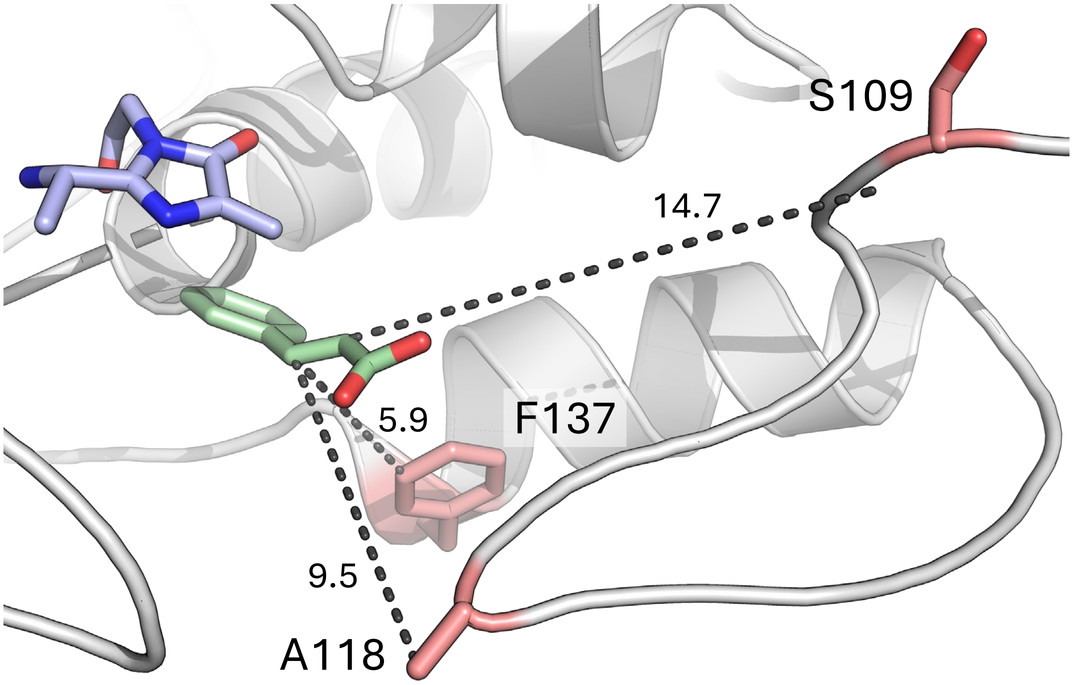

Fig. S2. Modeled active site of FxaPAL1 showing the MIO cofactor (blue), cinnamate ligand (green), and residues Ser109, Ala118, and Phe137, with distances (Å) to the ligand indicated. The protein backbone is from the AlphaFold-predicted FxaPAL1 structure; MIO and cinnamate are from the *Rhodobacter sphaeroides* tyrosine ammonia-lyase crystal structure (PDB: 2O78).
