# Supplementary material for: Building a directed evolution–genome editing pipeline for metabolic traits in specialty crop breeding
Source: Hortic Res. 2025 Oct 25;12(11):uhaf203. doi: 10.1093/hr/uhaf203 (PMC12574542; doi:10.1093/hr/uhaf203)
Supplement: Web_Material_uhaf203 [file web_material_uhaf203.zip › Supplementary Information.pdf]

Supplementary Information

PAL gene family and expression analysis

Transcriptome data of ripe fruits with six replicates were retrieved from European Nucleotide Archive [1]. RNAseq reads were aligned to the FaRR1 genome using Star v2.7 [2, 3]. Reads were counted by HTSEQ v.0.11 in the union mode with the 'nonunique none' flag [4]. PAL homologs in *Fragaria* × *ananassa* were identified using BLAST search and their expression in ripe fruits was compared in R.

FxaPAL1 sequence

The FxaPAL1 coding sequence was recoded for expression in *Escherichia coli* as follows.

```
ATGGCACTGAATGGTAATGGTAATTGCAATGGTGCAGCAGCAGTTGGTAGCTTTTGTCAAGGTGGTGGTCTGCTGCATGATCCGCT
GAATTGGAATCTGGCAGCAGAAGGTCTGAAAGGTAGCCATCTGGATGAACTGAAACGTATGGTTAACGATTATCGTAAACCGGTGG
TGATGTTAGGTGGTGAAAGCCTGACCGTTGGTCAGGTTGCAGCAATTGCAAACCATGATGGTGGTGTTCGTGTTGAACTGAGCGAA
GAAAAACGTGCCGGTGTTAAAGCAAGCAGCGATTGGGTTATGGATAGCATGGGTAAAGGCACCGATAGTTATGGTGTTACCACCGG
CTTTGGTGCAACCAGCCATCGTCGTACCAAAAATGGTGGTGCACTGCAGAAAGAACTGATTCGTTTTCTGAATGCCGGTATTTTTG
GTTCAAGCCTGGATAGCACCCATAAACTGCCGCATACCGCAACACGTGCAGCAATGCTGGTTCGCATTAATACCCTGCTGCAGGGC
TATAGCGGTATTCGTTTTGAAATTCTGGAAGCCATTACCAAACTGCTGAATGGCAATATTACCCCGTGTCTGCCGCTGCGTGGCAC
CATTAGCAGCAGCGGTGATCTGGTTCCGCTGAGCTATATTGCAGGTCTGCTGATTGGTCGTCCGAATAGCAAAAGCATTGGTCCGA
ATGGCGAAACCCTGACACCGGCAGAAGCATTTAAACTGGCAGGTATTGAAGGCGATTTCTTTGAACTGCAGCCGAAAGAAGGCCTG
GCACTGGTTAATGGCACCGCAGTTGGTTCAGGTATGGCAAGCGTTGTTCTGTTTGATGCAAATACCCTGGCAGTTCTGAGCGAAAT
TATGAGCGCAATTTTTGCAGAAGTGATGCAGGGTAAACCGGAATTTACCGATCATCTGACACATAAACTGAAACATCATCCGGGTC
AGATTGAAGCAGCAGCAATTATGGAACATATTCTGGATGGTAGCAGCTATGTTAAAGAAGCCAAAAAGGTTCACGAAATGGATCCG
CTGCAGAAACCGAAACAGGATCGTTATGCACTGCGTACCAGTCCGCAGTGGCTGGGTCCGCTGATTGAAGTTATTCGTAGCAGCAC
CAAAATGATCGAACGTGAAATTAACAGCGTGAATGATAATCCGCTGATCGATGTTAGCCGTAATAAAGCACTGCATGGTGGTAATT
TTCAGGGCACCCCGATTGGCACCGCCATGGATAATACCCGTCTGGCCCTGGCCAGCATTGGTAAACTGATTTTTGCACAGTTTAGC
GAGCTGGTGAACGATTTTTACAATAATGGTCTGCCGAGCAATCTGAGCGGTGGTAGCAATCCGAGCCTGGATTATGGTTTTAAAGG
TGCAGAAATTGCCATGGCCAGCTATTGTAGCGAATTACAGTTTCTGGCAAATCCGGTTACCAATCATGTTCAGAGCGCAGAACAGC
ATAATCAGGATGTTAATAGCCTGGGTCTGATTAGCAGCCGTAAAACCAGCGAAGCAGTTGATATTCTGAAACTGATGAGCAGCACC
TTTCTGGTTGCCCTGTGTCAGGCAGTTGATCTGCGTCACATGGAAGAAAATCTGAAAAGCGTTGTGAAAAACACCGTTAGCAAAGT
TGCACGTCGTACCCTGACCGTGGCGCTGAATGGTGAACTGCATCCGTCACGTTTTAGCGAAAAACATCTGCTGAGCGTTGTTGATC
GTGAATACCTGTTTAGCTATATCGATGATCCGTGTCTGGCAACCTATCCTCTGATGCAGAAACTGCGTGCAGAACTGGTTGAACAT
GCACTGAATAATGGCGATAAAGAGAAAAGCACCAACACCAGCATTTTTCTGAAAATCGCAGCCTTTGAAGAGGAACTGAAAAGTGT
TCTGCCTAAAGAAGTTGATAACGCACGCATGGAAATTGAAAATGGCAAAGCCGAAATTGCGAACCGTATTAAAGAATGTCGTAGCT
ATCCGCTGTATCGTTTTGTTCGTGAAGAACTGGGCACCAGCCTGCTGACCGGTGAGAAAATTCGTAGTCCGGGTGAAGAATGTGAT
AAAGTGTTTAATGCAATCTGCGCAGGCAAACTGATTGATCCTCTGCTGGAATGTCTGAAAGAATGGAATGGTGCACCGCTGCCGAT
TAGCTAA
```

Cloning of FxaPAL1 in pET-28b and generation of mutants

The codon-optimized FxaPAL1 sequence was synthesized by Twist Biosciences and amplified with primers AG005 and AG006 via the polymerase chain reaction with Q5® High-Fidelity DNA Polymerase (New England Biolabs). The pET-28b vector was amplified likewise with primers AG007 and AG008. The FxaPAL1 amplicon was cloned into pET-28b via Gibson assembly using the NEBuilder® HiFi DNA Assembly Kit (New England Biolabs) following the manufacturer's protocol, and transformed into *E. coli* TOP10 cells. The resulting plasmid (designated pET28b-FxaPAL1) was selected with kanamycin (50 µg ml^{-1}). The presence of the FxaPAL1 gene in transformants was verified by Sanger sequencing.

Mutants were generated with pET28b-FxaPAL1 as template and with the respective primer pairs using Q5® Site-Directed Mutagenesis Kit and Q5® High-Fidelity DNA Polymerase (New England Biolabs). Amplicons were transformed into *E. coli* TOP10 cells and sequence-verified.

Primer Name / Mutation	5' → 3' Sequence (lower case indicates the mutation)
AG005	CGCGGCAGCCATATGGCACTGAATGGTAATGGTAATTGC
AG006	TCGAGTGCGGCCGCAAGCTTAGCTAATCGGCAGC
AG007	TGCACCGCTGCCGATTAGCTAAGCTTGCGGCCGC
AG008	TGCAATTACCATTACCATTCAGTGCCATATGGCTGCCGC
F137H	ACTGATTCGTcatCTGAATGCCG<br>TCTTTCTGCAGTGCAC
S109I	AGGCACCGATattTATGGTGTTAC<br>TTACCCATGCTATCCATAAC
A118G	CGGCTTTGGTggcACCAGCCATC<br>GTGGTAACACCATAACTATCG

Expression and extraction of recombinant FxaPAL1 enzymes

E. coli BL21(*DE3*) was transformed with pET28b-FxaPAL1 plasmid DNA of each mutant. Individual transformed colonies were grown in 3 ml of Luria Bertani medium (LB) plus kanamycin 50 μg ml^{-1} and incubated overnight at 37°C, 250 rpm. These precultures were used to inoculate 100 ml of LB plus kanamycin 50 μg ml^{-1} (starting OD_{600nm} = 0.05). The expression cultures were grown at 37°C in 500-ml Erlenmeyer flasks with shaking at 250 rpm until OD_{600nm} reached 0.4-0.6. The flasks were then cooled on ice for 20 min and protein expression was induced by adding isopropyl β-D-1-thiogalacto-pyranoside (IPTG, final concentration 0.5 mM). The cultures were then held at 19°C, 180 rpm for 18 h. Cells were harvested by centrifugation (4,000 g, 10 min, 4°C) and the pellet was resuspended in 2.5 ml of lysis buffer (100 mM Tris-HCl, pH 8.5, 200 mM NaCl). The suspension was sonicated on ice for 9 cycles (60% power, 15 s on, 45 s off) and cleared by centrifugation (25,000 g, 40 min, 4°C). The supernatant was diluted with exchange buffer (100 mM Tris-HCl, pH 8.5) to a volume of 15 ml and transferred to an Amicon® Ultra-15 Centrifugal Filter Unit (10 kDa cutoff). After centrifuging (5,000 g, 1 h, 4°C) to concentrate to 500 μl, 15 ml of exchange buffer was added and the sample was reconcentrated to 500 μl. Samples were stored at -20°C after adding glycerol (final concentration 20%).

Protein estimation

Total protein concentration was measured using the Bio-Rad Protein Assay kit with bovine serum albumin as the standard. Recombinant FxaPAL1 protein was analyzed by SDS-PAGE; gels were stained with GelCode™ Blue Safe Protein Stain (Thermo Fisher Scientific) and the FxaPAL1 band was quantified (as percent of total protein in the extract) using ImageJ software.

Spectrophotometric PAL and TAL assays

Assays (final volume 100 µl) were run in quartz cuvettes at room temperature (~22°C) in 100 mM Tris-HCl. PAL assays contained 0.8 µg total protein and 10 to 3000 µM L-phenylalanine; cinnamate formation was monitored at 290 nm (ε = 8996 $M^{-1}cm^{-1}$). TAL assays contained 3.5 µg total protein and 10 to 1800 µM L-tyrosine; 4-coumarate formation was monitored at 315 nm (ε = 13787 $M^{-1}cm^{-1}$). Blanks were run without substrate; the small rate of absorbance change was used to correct assay data. The molar absorption coefficients of cinnamate and 4-coumarate are calculated from calibration curves at 290 nm and 315 nm, respectively.

Supplementary References

1. Sánchez-Sevilla JF, Vallarino JG, Osorio S *et al*. Gene expression atlas of fruit ripening and transcriptome assembly from RNA-seq data in octoploid strawberry (*Fragaria* × *ananassa*). *Sci Rep*. 2017;**7**:1–13.
2. Dobin A, Davis CA, Schlesinger F *et al*. STAR: ultrafast universal RNA-seq aligner. *Bioinformatics*. 2013 **29**:15–21.
3. Hardigan MA, Feldmann MJ, Pincot DD *et al*. Blueprint for phasing and assembling the genomes of heterozygous polyploids: application to the octoploid genome of strawberry. *bioRxiv*. 2021; doi 10.1101/2021.11.03.467115.
4. Anders S, Pyl PT, Huber, W. HTSeq – a Python framework to work with high-throughput sequencing data. *Bioinformatics.* 2015;**31**:166–69.
