# Supplementary material for: Building a directed evolution–genome editing pipeline for metabolic traits in specialty crop breeding
Source: Hortic Res. 2025 Oct 25;12(11):uhaf203. doi: 10.1093/hr/uhaf203 (PMC12574542; doi:10.1093/hr/uhaf203)
Supplement: Web_Material_uhaf203 [file web_material_uhaf203.zip › Table S1.pdf]

Table S1. Kinetic parameters of recombinant FxaPAL1 enzymes.

Measurements were made on *E. coli* soluble protein extracts in which FxaPAL1 accounted for 10–12% of the total protein. The percentage of FxaPAL1 protein in each extract was estimated by scanning SDS-PAGE gels and used to calculate k_{cat} values from the total amount of protein added to assays.

Protein	PAL activity			TAL activity		
	K_M (μM)	k_{cat} (s^{-1})	k_{cat}/K_M (s^{-1} mM^{-1})	K_M (μM)	k_{cat} (s^{-1})	k_{cat}/K_M (s^{-1} mM^{-1})
FxaPAL1	37 ± 1.2	1.6 ± 0.03	44 ± 1.4	ND	ND	ND
FxaPAL1^{S109I}	40 ± 2.6	0.03 ± 0.001	0.8 ± 0.03	1010 ± 39	0.05 ± 0.0008	0.05 ± 0.013
FxaPAL1^{A118G}	43 ± 1.5	1.0 ± 0.005	24 ± 0.9	140 ± 27	0.002 ± 0.0003	0.02 ± 0.002
FxaPAL1^{F137H}	461 ± 51	1.2 ± 0.05	2.7 ± 0.2	120 ± 20	0.02 ± 0.0004	0.21 ± 0.002
FxaPAL1$^{F137H\text{-}A118G}$	2210 ± 198	0.8 ± 0.08	0.4 ± 0.01	482 ± 220	0.02 ± 0.002	0.13 ± 0.01
FxaPAL1$^{F137H\text{-}S109I}$	43 ± 6.2	0.8 ± 0.02	20 ± 2.8	31 ± 1	0.02 ± 0.0003	0.71 ± 0.02
FxaPAL1$^{F137H\text{-}S109I\text{-}A118G}$	98 ± 4.0	1.2 ± 0.008	13 ± 0.4	60 ± 3.5	0.04 ± 0.0007	0.63 ± 0.03

ND: not determined. The data represent the mean of three technical replicates ± SE.
