# Supplementary material for: Building a directed evolution–genome editing pipeline for metabolic traits in specialty crop breeding
Source: Hortic Res. 2025 Oct 25;12(11):uhaf203. doi: 10.1093/hr/uhaf203 (PMC12574542; doi:10.1093/hr/uhaf203)
Supplement: Web_Material_uhaf203 [file web_material_uhaf203.zip › Table S2.pdf]

Table S2. Estimates of the expression level of S109I–F137H mutant FxaPAL1 and of its possible DE-improved versions that would be needed to supply NH_3 to support a yeast cell doubling time of 24 h if the TAL activity of the mutant FxaPAL1 is the sole source of nitrogen.

Note that the k_{cat} value of the TAL activity of the S109I–F137H mutant (0.022 s^{-1}) could in principle be increased several thousand-fold by DE. This would reduce the amount of enzyme required to <0.05% of total cell protein, which is within the range achievable in the OrthoRep system [37].

**Parameter**	**Value**	**Source**
Mutant FxaPAL1 molecular mass	77.9 kDa	
Tyrosine substrate k_{cat}	0.022 s^{-1} (79 h^{-1})	This study
Reported k_{cat} for natural TAL enzymes	3.4 s^{-1}<br>27.7 s^{-1}<br>114 s^{-1} (at 40°C)	Watts et al., 2006<br>Kyndt et al., 2002<br>Zhou et al, 2016
Yeast culture dry weight at OD_{600} = 1	0.62 mg ml^{-1}	Bionumbers database
Yeast cell total protein content	40% of dry weight	Bionumbers database
Nitrogen content of protein	Protein/6.25	Mariotti et al., 2008
Minimum doubling time for OrthoRep	24 h	García-García et al., 2022
Total protein content of culture at OD_{600} = 1	**248 µg ml^{-1}**	
N content of culture at OD_{600} = 1	2.8 µmol ml^{-1}	
NH_3 from tyrosine required per hour	120 nmol h^{-1}	
Mutant FxaPAL1 needed to supply NH_3	1.5 nmol ml^{-1} = **120 µg ml^{-1}** = 48% of total protein	
Increases in mutant FxaPAL1 k_{cat} needed to match those of natural TAL enzymes above	340×<br>2,800×<br>5,700× (assuming Q_{10} = 2)	
Mutant FxaPAL1 needed to supply NH_3 if DE increases k_{cat} by:<br>340×……………………………………………..<br>2,800×…………………………………………...<br>5,700×…………………………………………...	 % of total protein<br>0.17%<br>0.02%<br>0.01%	
